# Supplementary material for: Snap evaporation of droplets on smooth topographies
Source: Nat Commun. 2018 Apr 11;9:1380. doi: 10.1038/s41467-018-03840-6 (PMC5895805; doi:10.1038/s41467-018-03840-6)
Supplement: Supplementary file 1 — Supplementary Information [file 41467_2018_3840_MOESM1_ESM.pdf]

Snap evaporation of droplets on smooth topographies
Supplementary Information

Wells et al.

SUPPLEMENTARY METHODS

Supplementary Note 1: Preparation of liquid-impregnated rough surfaces (LIRS)

Surface topographies were designed using standard 3D computer modelling software (SolidWorks). Samples of dimensions 5 cm × 5 cm × 0.5 cm were produced using an Objet30 3D printer in Vero White Plus RGD835 resin.

Planar-wave surfaces were modelled using a local elevation

$$\eta(x) = \epsilon \cos\left(\frac{2\pi x}{\lambda}\right), \tag{1}$$

where x is a lateral coordinate (see Supplementary Figure 1a). Surfaces were produced for three different values of the amplitude, $\epsilon = \{50, 100, 200\}$ μm, and a fixed wavelength, $\lambda = 2$ mm.

Egg-box surfaces were modelled using a local elevation

$$\eta(x, y) = \epsilon \cos\left(\frac{2\pi x}{\Lambda}\right) \cos\left(\frac{2\pi y}{\Lambda}\right), \tag{2}$$

where $\epsilon = 200$ μm and $\Lambda = 4$ mm. The diagonal wavelength for this pattern is $\lambda = \Lambda/\sqrt{2} \approx 2.83$ mm (see Supplementary Figure 1b).

Once printed, each sample was spray-coated five times using a superhydrophobic nano-coating (Glaco Mirror Coat, Soft 99 Co.) and then baked at 80°C for 20 minutes between coats. The surfaces were subsequently immersed in a bath of the silicone oil (Sigma-Aldrich, CAS No. 378348) and withdrawn vertically at a controlled rate of 0.1 mm s^{-1} using a robot (Fisnar F4200N).

Supplementary Note 2: Surface characterisation

Contact angle measurements

Contact angle measurements for water droplets on flat LIRS were carried out using a computer-assisted contact angle meter (Krüss DSA30). A 4-μL de-ionized water droplet was placed on a levelled surface. The profile of the droplet was calculated using the Krušs Drop Shape Analysis (DSA) software (see Supplementary Figure 2). The software fits tangent lines (red lines in the figure) at the points where the liquid gas interface (solid red curve) intersects the solid surface (green horizontal line). The contact angles at the left and right edges of the droplet are calculated from the slope of the tangents. The average apparent contact angle based on 10 measurements is $\theta_a = 109° \pm 0.4°$, where the uncertainty is a standard deviation of the sample.

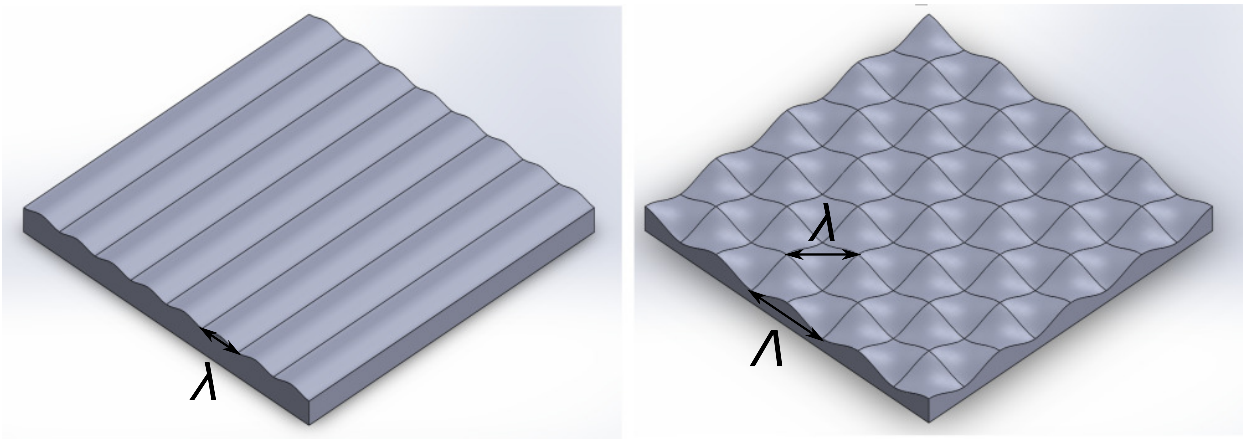

(a) (b)

Supplementary Figure 1: Computer generated models of (a) planar-wave and (b) egg-box surface topographies.

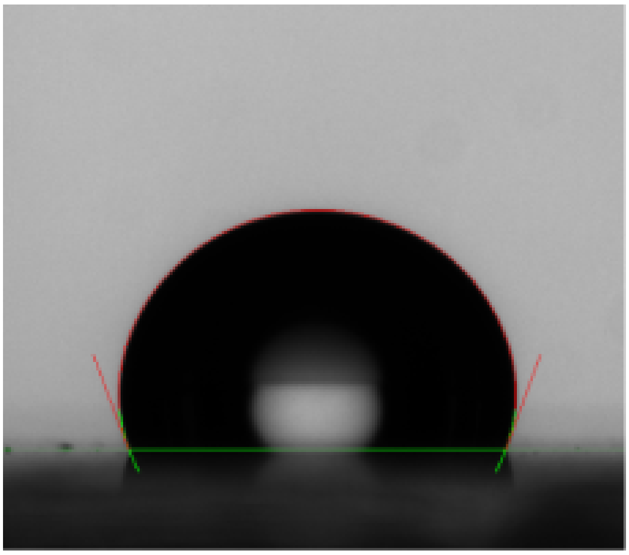

Supplementary Figure 2: Illustration of drop shape analysis. The image shows the contour and intersection tangents to the interface, shown as a red curve and red straight lines, used to determine the apparent contact angle with the solid surface (horizontal green line). The droplet volume is 4 μL.

Sliding angle measurements

Droplet sliding-angle measurements where carried out using a computer-assisted contact angle meter equipped with a calibrated tilt stage (Krüss DSA30). De-ionized water droplets (volume = 4 μL) were first dispensed on a flat LIRS substrate resting on a levelled stage (see Supplementary Figure 3). The tilt angle of the stage was varied at 0.1° increments. The sliding angle was

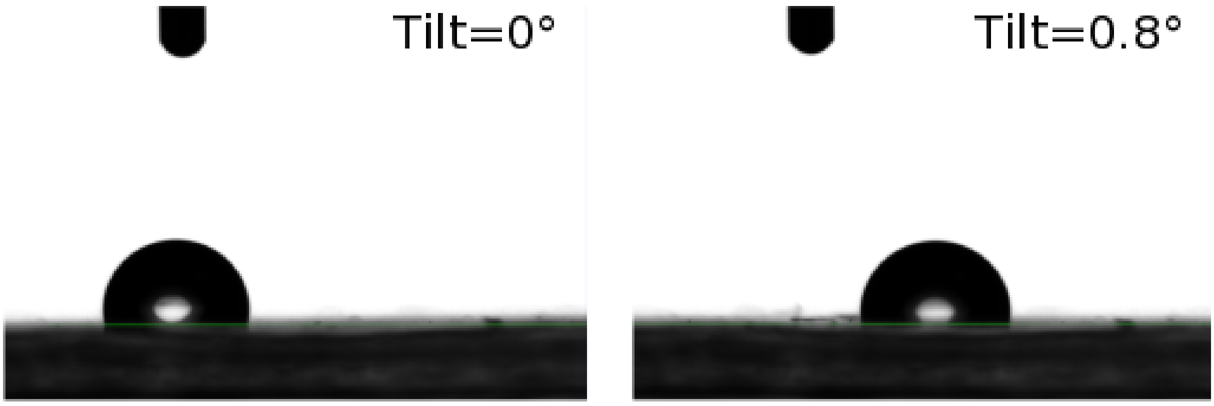

(a) (b)

Supplementary Figure 3: Measurement of the sliding angle on a flat LIRS. (a) Stationary droplet on a levelled surface. (b) Moving droplet on a surface at a tilt angle of 0.8°. The measured tilt angle of the surface is indicated in each panel.

recorded as the first tilt angle for which the droplet moved continuously. The measured sliding angle recorded is $\theta_s = 0.75° \pm 0.05°$, where the uncertainty is half of the minimum precision provided by the experimental apparatus.

Supplementary Note 3: Evaporation experiments

Evaporation on flat surfaces

A freshly prepared flat LIR surface was placed in a controlled humidity chamber connected to a contact angle meter system. Droplets of initial volume 80 μL were dispensed using a micro-syringe onto the centre of the sample. The relative humidity of the chamber was set to 20% with a typical variation during the experiment of $1-5\%$. The base radius and apparent contact angle of the droplet was tracked using the DSA software detailed in Supplementary Note 2. The experiment was repeated 5 times.

Evaporation on planar-wave surfaces

A freshly prepared planar-wave LIRS surface was placed on a levelled stage (see Supplementary Figure 4). A de-ionized water droplet (volume = 80 μL) was manually deposited onto the centre of

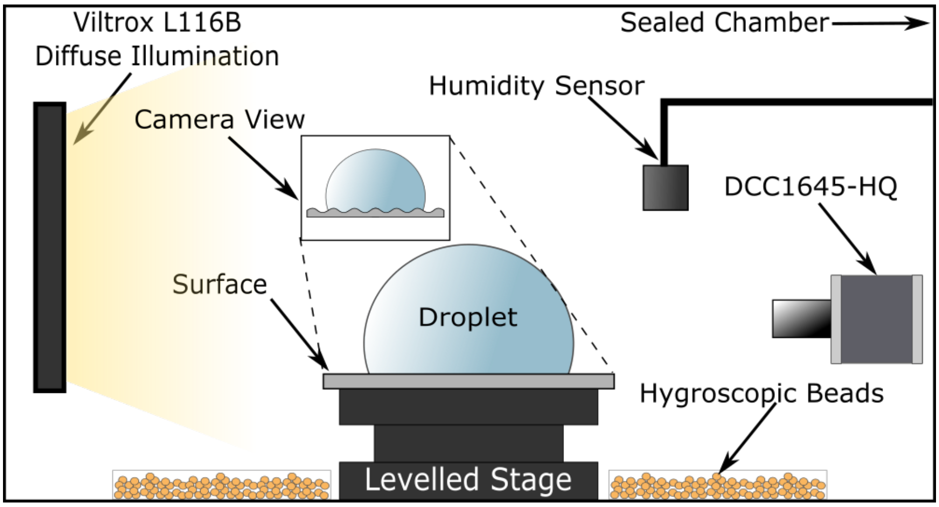

Supplementary Figure 4: Schematic of the experimental setup of a droplet evaporating on a planar-wave LIR surface.

the surface using a micro-syringe. The droplet was allowed to evaporate within a closed chamber. The humidity of the chamber was controlled by using hygroscopic silica beads. The temperature and relative humidity within the chamber were tracked using a digital sensor (Adafruit HTU21DF), and ranged between 20-25° C, and 20-30%, respectively. Under such conditions, the typical evaporation time for an 80-μL droplet is 5 hours, giving an average evaporation rate of 4 nL s^{-1}. Front images of the droplet were captured using a time-lapse camera (Thorlabs DCC1645-HQ) with diffuse backlight to enhance visibility (Viltrox L116B). Time-lapse images were recorded at a rate of 1 frame every 300 seconds. Base radius and apparent contact angle measurements were obtained from the time-lapse images by fitting a tangent line to the droplet's profile at the intersection with the solid surface, and calculating its orientation relative to the horizontal. Three experiments were carried out for each sample.

Evaporation on egg-box surfaces

An egg-box LIRS surface was placed on a levelled stage (see Supplementary Figure 5a). A de-ionized water droplet (volume = 0.5 mL) was manually deposited onto the centre of the surface using a micro-syringe. The droplet was allowed to evaporate under ambient temperature and humidity conditions. During evaporation, a top view of the droplet was recorded using a camera system (Allied Vision Mako U130B) and two diffuse panel lights (Viltrox L116B) placed on either side of the droplet to enhance visibility. Time-lapse images were obtained using a bespoke Lab-

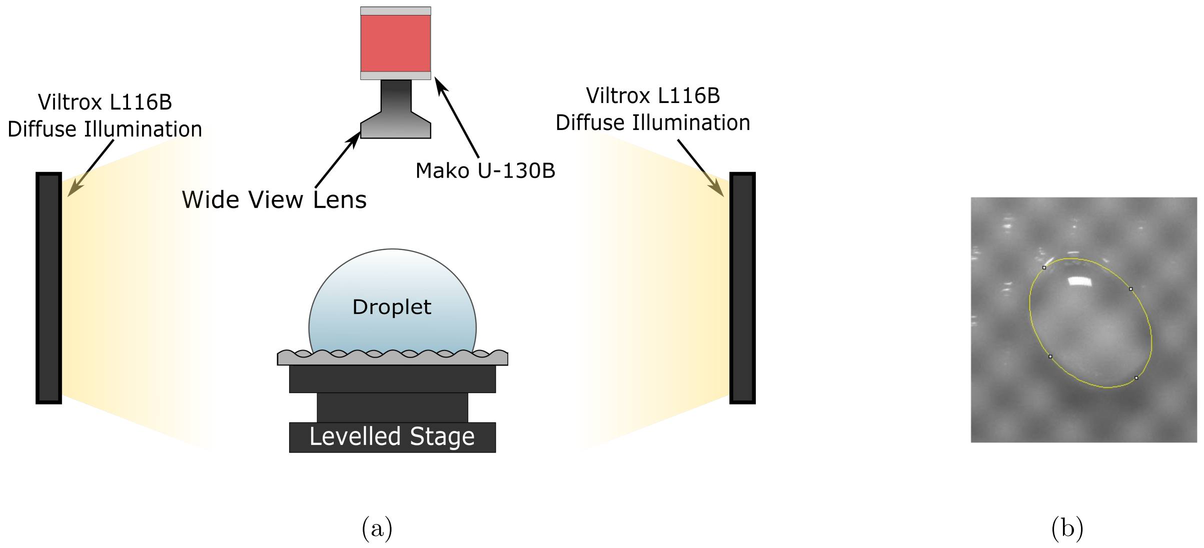

Supplementary Figure 5: Evaporation on egg-box LIR surfaces. (a) Schematic of the experimental setup. (b) Illustration of the analysis of the droplet's apparent contact line shape. The yellow line corresponds to a fitted ellipsoid.

VIEW program at 1 frame every 300 seconds. The height and width of the droplet were measured by using the best fit of an ellipse to the apparent edge of the droplet (see Supplementary Figure 5b). The experiment was repeated five times.

Supplementary Note 4: Lattice-Boltzmann simulations

Simulations were carried out using a binary-fluid lattice-Boltzmann algorithm. The geometry of the lattice is a cubic grid where lattice nodes are connected to their zeroth, first and second nearest neighbours (so-called D2Q9 and D3Q15 models for 2D and 3D simulations, respectively) [1]. Lattice nodes can be either "solid" or "fluid". At any given fluid node, indicated by a position vector $\mathbf{r}$, we define two probability distribution functions, f_i and g_i, where the index i refers to the advection lattice propagation directions, $\mathbf{c}_i$. The time evolution of the distribution functions is given by the single-relaxation-time lattice-Boltzmann equations, $f_i(\mathbf{r}+\mathbf{c}_i, t+1) = f_i(\mathbf{r}, t) + (f_i - f_i^{\mathrm{eq}})/\tau_f$ and $g_i(\mathbf{r}+\mathbf{c}_i, t+1) = g_i(\mathbf{r}, t) + (g_i - g_i^{\mathrm{eq}})/\tau_g$. These include a collision step where the distribution functions relax towards equilibrium values, indicated by the superscript "eq" over the respective relaxation timescales τ_f and τ_g (set to unity in the simulations), followed by a propagation step where the f_i and g_i are advected to their neighbouring sites. The macroscopic hydrodynamic fields are recovered through the moments of the distribution functions, i.e., the density $\rho = \sum_i f_i$, momentum density $\rho\mathbf{v} = \sum_i \mathbf{c}_i f_i$, and a 'phase field' $\phi = \sum_i g_i$ which labels two binary fluid

phases. The instantaneous hydrodynamic fields determine the pressure and chemical potential of the fluid mixture via a Cahn-Hilliard free-energy model. The hydrodynamic behaviour of the fluid, in turn, is governed by the macroscopic variables via collisions of the distribution functions. A suitable choice of the equilibrium distribution functions recovers the macroscopic equations of motion of the fluid in the limit of small Mach numbers. This is done such that the first moments read, $\sum_i f_i^{\rm eq} = \rho$, $\sum_i \mathbf{c}_i f_i^{\rm eq} = \rho\mathbf{v}$, $\sum_i \mathbf{c}_i\mathbf{c}_i f_i^{\rm eq} = P + \rho\mathbf{v}\mathbf{v}$, $\sum_i g_i^{\rm eq} = \phi$, $\sum_i \mathbf{c}_i g_i^{\rm eq} = \phi\mathbf{v}$, and $\sum_i \mathbf{c}_i\mathbf{c}_i g_i^{\rm eq} = M\mu/(\tau_g - 1/2) + \phi\mathbf{v}\mathbf{v}$, where P is the pressure tensor, and μ is the chemical potential [2]. After the collision step is performed, external forces can be applied to include gravitational effects.

We consider two types of boundary conditions: *no-slip* boundary conditions, applied at the solid-fluid interface, and *open* boundaries that allow evaporation fluxes exit the simulation domain. The no-slip boundary conditions are enforced using a bounce-back algorithm [3] enhanced with a linear interpolation scheme to model the curved boundaries [4]. This ensures that no flows permeate through the solid. To model the wetting properties of the fluids, the derivatives of the phase-field are subject to a Neumann boundary condition [5]. Explicitly, the boundary conditions at any point on the solid surface (denoted by the position vector $\mathbf{r}_{\rm w}$) read

$$\rho\mathbf{v}(\mathbf{r}_{\rm w}) = 0, \tag{3}$$

$$\mathbf{n}\cdot\nabla\mu(\mathbf{r}_{\rm w}) = 0, \tag{4}$$

$$\mathbf{n}\cdot\nabla\phi(\mathbf{r}_{\rm w}) = -\frac{\sqrt{8}h}{3\gamma\xi}, \tag{5}$$

where $\mathbf{n}$ is a vector normal to the solid, h is a parameter that controls the equilibrium contact angle, γ is the fluid-fluid surface tension and ξ is the fluid-fluid interface thickness.

Supplementary Equations (3)–(5) allow the pinning-free motion of the contact lines by means of diffusive fluxes of the chemical potential along the solid surface. Such a mechanism produces an apparent slip-length and a dynamic contact angle that deviates from its equilibrium value [6–8], in a manner consistent with contact-line hydrodynamics [9, 10].

The open boundaries are prescribed with a zeroth-order anti-bounceback algorithm [4] by specifying

$$\mathbf{n}\cdot\nabla\phi(\mathbf{r}_{\rm o}) = 0, \tag{6}$$

$$\mathbf{n}\cdot\nabla\cdot P(\mathbf{r}_{\rm o}) = 0 \tag{7}$$

$$\mu(\mathbf{r}_{\rm o}) = \mu_{\rm b}, \tag{8}$$

for all $\mathbf{r}_{\rm o}$ on the top plane of the simulation domain where the open boundary is specified. Supple-

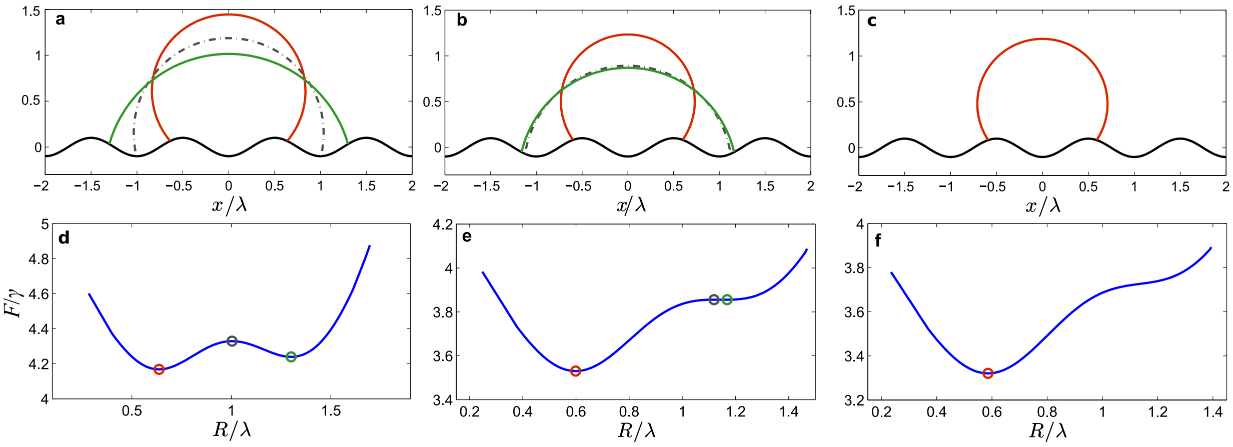

Supplementary Figure 6: Examples of droplet solutions on a valley, $\tilde{x} = 0$, with $A/\lambda^2 = 2$ (**a**), 1.5 (**b**), and 1.35 (**c**). In all cases the equilibrium contact angle is $\theta_{\rm e} = 105°$ and the surface aspect ratio is $\epsilon/\lambda = 0.1$. Bottom panels (d,e,f) show their corresponding non-dimensional interfacial energy F/γ as function of the base radius where circles mark the fixed points. A local stability analysis reveals that the solutions shown as solid lines in panels (a,b,c) are stable configurations to δR pertubations while the grey dot-dashed lines are unstable. Furthermore, the solution shown as green solid line is stable against δl perturbations in panel **a** but unstable against δl perturbations in panel **b**.

mentary Equations (6) and (7) ensure that the flow is driven purely by diffusive flows. Evaporation is caused by fixing μ_b to an out-of-equilibrium value [11].

We set the simulation parameters, in lattice-Boltzmann units, to the following values: density $\rho = 1$, viscosity $\nu = 1/6$, surface tension $\gamma = 10^{-3}$, mean contact angle $\theta_{\rm e} = 105°$, contact angle variance $\sigma(\theta_{\rm e}) = 4°$ for the chemical noise in the solid surface, and the value of the chemical potential at the open boundaries $\mu_{\rm b} = -10^{-5}$, which is sufficient to drive the evaporation of the droplets in the diffusion-limit regime [11]. The 3D simulation domain is contained in a box of size $120 \times 120 \times 80$, the mobility $M = 1$, interface thickness $\xi = 4$, and gravitational acceleration $\mathbf{g} = -2 \times 10^{-6}\hat{\mathbf{z}}$. The 2D simulations were carried out in a box of size 240×120, and parameters: $M = 4$, $\xi = 6$, and $\mathbf{g} = 0$. As an initial condition for all simulations, a spherical droplet of radius 1.5λ was centred at either a peak or a valley. The simulations were run for $\sim 10^7$ iterations, which was a sufficiently long simulation time for the droplet to evaporate completely from the solid surface.

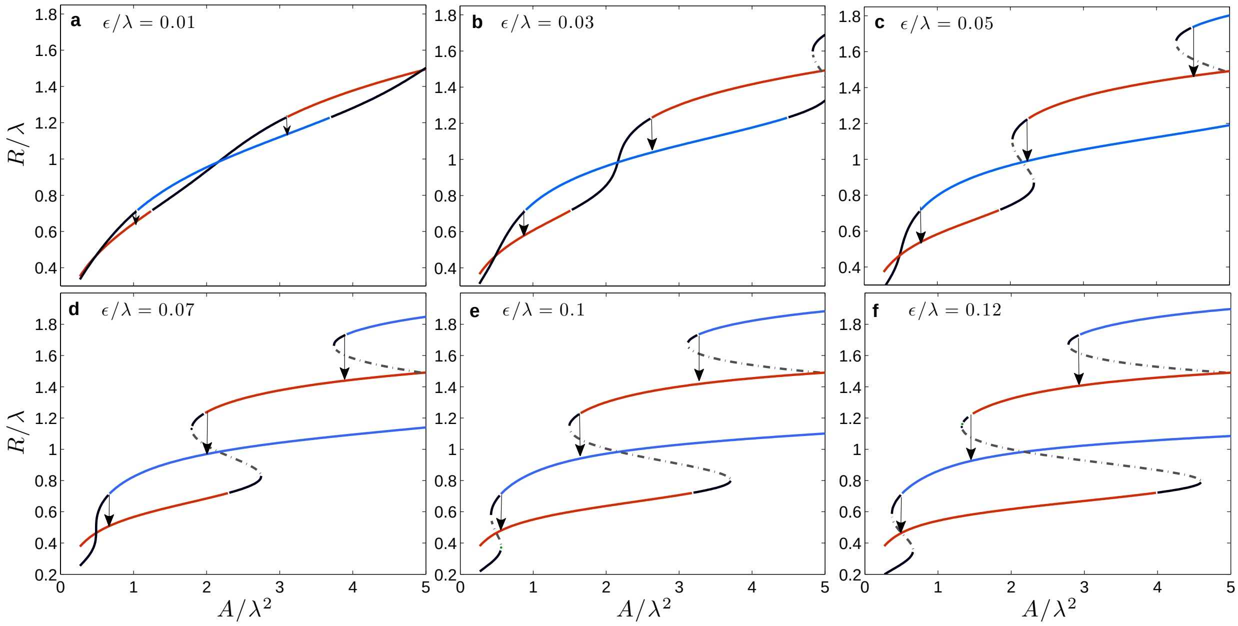

Supplementary Figure 7: Bifurcation diagrams of the base radius of the droplet for different surface aspect ratio ϵ/λ; **a,** $\epsilon/\lambda = 0.01$, **b,** $\epsilon/\lambda = 0.03$, **c,** $\epsilon/\lambda = 0.05$, **d,** $\epsilon/\lambda = 0.07$, **e,** $\epsilon/\lambda = 0.1$, and **f,** $\epsilon/\lambda = 0.12$. Blue and red branches correspond to stable points on peak and valley configurations, respectively, black solid lines correspond to saddle nodes where the droplet is stable to axisymmetric perturbations but stable to asymmetric perturbations. Grey dot-dashed branches correspond to unstable solutions. Arrows mark the point where the droplet will snap laterally and shift to the next available stable configuration (either peak or valley). In all cases the equilibrium contact angle is $\theta_\mathrm{e} = 105°$.

Supplementary Note 5: Two-dimensional theory

Equilibrium properties

We consider a two-dimensional (2D) sessile droplet on a smooth solid substrate which has a sinusoidal topography given by:

$$\eta(x) = -\epsilon \cos(kx), \tag{9}$$

where ϵ is the amplitude, and $k = 2\pi/\lambda$ with λ the wavelength. In equilibrium, the shape of the droplet, $h(x)$, is given by the Young-Laplace equation:

$$\frac{\Delta p}{\gamma} = \frac{y_{xx}}{(1 + y_x^2)^{3/2}}, \tag{10}$$

where $y(x) = h(x) + \eta(x)$, Δp is the pressure difference across the liquid-gas interface and γ is its surface tension. For a droplet located either on a valley (with midpoint $\tilde{x} = x_\mathrm{V} = 0, \pm\lambda, \pm 2\lambda, \dots$)

or a peak ($\tilde{x} = x_P = \pm\frac{1}{2}\lambda, \pm\frac{3}{2}\lambda, \dots$), whose contact points are at $x = \tilde{x} \pm R$, where R is the base radius, the apparent contact angle is [12]:

$$\theta_a = \theta - \tan^{-1}\left[\epsilon k \cos k\tilde{x} \sin kR\right], \tag{11}$$

where θ is the contact angle, which at equilibrium corresponds to the equilibrium contact angle θ_e. The area A of the droplet is then given by

$$A = \frac{R^2}{2}\left(\frac{2\theta_a - \sin 2\theta_a}{\sin^2\theta_a}\right) + 2\epsilon R \cos k\tilde{x}\left(\frac{\sin kR}{kR} - \cos kR\right). \tag{12}$$

For a given area, we numerically solve the above equation and find the different equilibrium configurations with $\theta = \theta_e$. Supplementary Figures 6a , b and c show examples of the different droplet shapes found at a valley ($\tilde{x} = 0$) for $A/\lambda^2 = 2$, 1.5 and 1.35. In all cases, the equilibrium contact angle is $\theta_e = 105°$.

Local stability analysis

We study the local stability of the equilibrium solutions by analysing the interfacial energy which is given by:

$$F(R, \tilde{x}) = \gamma\frac{2\theta_a R}{\sin\theta_a} - \cos\theta_e \int_{\tilde{x}-R}^{\tilde{x}+R} \sqrt{1 + (\epsilon k)^2 \sin^2(kx)}\, dx. \tag{13}$$

By locally perturbing the equilibrium points with axisymmetric perturbations ($R \to R + \delta R$), and asymmetric perturbations ($\tilde{x} \to \tilde{x} + \delta\tilde{x}$), we can construct, for the fixed area given by Supplementary Equation (12), the energy landscape $F(R, \tilde{x})$. We find that the equilibrium configurations correspond to extrema of the energy function, for which $\partial_{\tilde{x}} F = \partial_R F = 0$, and can be a local minimum (stable to both δR and $\delta\tilde{x}$), a local maximum (unstable to both δR and $\delta\tilde{x}$), or a saddle node (stable to δR perturbations but unstable to $\delta\tilde{x}$ perturbations). Supplementary Figures 6d, e, and f show plots of the energy as function of R/λ for the three cases of A considered in Supplementary Figures 6a, b, and c, where we can see how equilibrium solutions emerge as A is varied.

Therefore, by continuously changing the droplet area we can construct the bifurcation diagrams for a droplet on a valley or peak of the solid surface. The results for different surface aspect ratios are shown in Supplementary Figure 7a-f. We can see that for small aspect ratios and small droplet areas, the stability of the equilibrium solutions alternates between saddle nodes (black solid lines) and stable points (blue/red solid lines for peak/valley solutions). The transition between these sates is a consequence of a pitchfork bifurcation where two additional saddle node branches emerge

on the $(\tilde{x}, A)$ plane. As we increase the surface aspect ratio, we observe the emergence of fold bifurcations (see e.g. Supplementary Figure 7c) giving rise to unstable solutions (grey dot-dashed lines).

SUPPLEMENTARY REFERENCES

[1] Lee, T. & Lin, C.-L. A stable discretization of the lattice Boltzmann equation for simulation of incompressible two-phase flows at high density ratio. *J. Comput. Phys.* **206**, 16–47 (2005).

[2] Swift, M. R., Orlandini, E., Osborn, W. & Yeomans, J. Lattice Boltzmann simulations of liquid-gas and binary fluid systems. *Phys. Rev. E* **54**, 5041 (1996).

[3] Bouzidi, M., Firdaouss, M. & Lallemand, P. Momentum transfer of a Boltzmann-lattice fluid with boundaries. *Phys. Fluids* **13**, 3452–3459 (2001).

[4] Ginzburg, I., Verhaeghe, F. & d'Humieres, D. Two-relaxation-time lattice Boltzmann scheme: About parametrization, velocity, pressure and mixed boundary conditions. *Commun. Comput. Phys.* **3**, 427–478 (2008).

[5] Cahn, J. W. Critical point wetting. *J. Chem. Phys.* **66**, 3667–3672 (1977).

[6] Jacqmin, D. Contact-line dynamics of a diffuse fluid interface. *J. Fluid Mech.* **402**, 5788 (2000).

[7] Ding, H. & Spelt, P. D. M. Inertial effects in droplet spreading: a comparison between diffuse-interface and level-set simulations. *J. Fluid Mech.* **576**, 287–296 (2007).

[8] Ding, H. & Spelt, P. D. M. Wetting condition in diffuse interface simulations of contact line motion. *Phys. Rev. E* **75**, 046708 (2007).

[9] Briant, A. J. & Yeomans, J. M. Lattice Boltzmann simulations of contact line motion. II. Binary fluids. *Phys. Rev. E* **69**, 031603 (2004).

[10] Kusumaatmaja, H., Hemingway, E. J. & Fielding, S. M. Moving contact line dynamics: from diffuse to sharp interfaces. *J. Fluid Mech.* **788**, 209–227 (2015).

[11] Ledesma-Aguilar, R., Vella, D. & Yeomans, J. M. Lattice-Boltzmann simulations of droplet evaporation. *Soft Matter* **10**, 8267–8275 (2014).

[12] Huh, C. & Mason, S. Effects of surface roughness on wetting (theoretical). *J. Colloid Interface Sci.* **60**, 11–38 (1977).
