# Supplementary material for: Snap evaporation of droplets on smooth topographies
Source: Nat Commun. 2018 Apr 11;9:1380. doi: 10.1038/s41467-018-03840-6 (PMC5895805; doi:10.1038/s41467-018-03840-6)
Supplement: Supplementary file 3 — Description of Additional Supplementary Files [file 41467_2018_3840_MOESM3_ESM.pdf]

Description of Additional Supplementary Files

File Name: Supplementary Movie 1
Description: Time-lapse video of an 80-μL droplet evaporating on a planar wave LIRs surface of wavelength λ = 2 mm and amplitude ε = 200 μL.

File Name: Supplementary Movie 2
Description: Comparison of a 3D lattice-Boltzmann simulation to an experimental time-lapse sequence. In both videos, the total video time is normalised using the evaporation time.

File Name: Supplementary Movie 3
Description: 2D lattice-Boltzmann simulation of a droplet evaporating on a wavy surface in the presence of a static surface noise.

File Name: Supplementary Movie 4
Description: 2D lattice-Boltzmann simulation of a droplet evaporating on a noise-free wavy surface.
